# Long-term exposure to ambient air pollutants and mental health status: A nationwide population-based cross-sectional study

**DOI:** 10.1371/journal.pone.0195607

**Published:** 2018-04-09

**Authors:** Jinyoung Shin, Jin Young Park, Jaekyung Choi

**Affiliations:** 1 Department of Family Medicine, Research Institute of Medical Science, Konkuk University School of Medicine, Konkuk University Medical Center, Seoul, South Korea; 2 Department of Psychiatry, Gangnam Severance Hospital, Yonsei University College of Medicine, Seoul, South Korea; Chiba Daigaku, JAPAN

## Abstract

There is a suspected but unproven association between long-term exposure to ambient air pollution and mental health. The aim of this study is to investigate the association between long-term exposure to ambient air pollution and subjective stress, depressive disorders, health-related quality of life (QoL) and suicide. We selected 124,205 adults from the Korean Community Health Survey in 2013 who were at least 19 years old and who had lived in their current domiciles for > five years. Based on the computer-assisted personal interviews to measure subjective stress in daily life, EuroQoL-5 dimensions, depression diagnosis by a doctor, suicidal ideation, and suicidal attempts, we evaluated the risk of mental disorders using multiple logistic regression analysis according to the quartiles of air pollutants, such as particulate matter <10μm (PM_10_), nitrogen dioxide (NO_2_), carbon monoxide (CO), and sulfur dioxide, using yearly average concentration between August 2012 and July 2013. The prevalence of high stress, poor QoL, depressiveness, diagnosis of depression, and suicide ideation was positively associated with high concentrations of PM_10_, NO_2_, and CO after adjusting for confounding factors. Men were at increased risk of stress, poor QoL, and depressiveness from air pollution exposure than were women. The risk of higher stress or poor QoL in subjects < age 65 increased with air pollution more than did that in subjects ≥ age 65. Long-term exposure to ambient air pollution may be an independent risk factor for mental health disorders ranging from subjective stress to suicide ideation.

## Introduction

Ambient air pollution is composed of a heterogeneous mixture of compounds, including particulate matter (PM), nitrogen dioxide (NO_2_), carbon monoxide (CO), and sulfur dioxide (SO_2_). These particles are composed of both solid and liquid components that originate from multiple sources, including vehicle exhaust, road dust, and windblown soil [1].

A growing body of evidence indicates that elevated levels of air pollution are associated with mental disorders such as depression and suicide. Elderly subjects experienced the aggravation of their depressive symptoms after 3-day exposure to air pollutants [2]. Emergency department visits for depressive episode were associated with increased levels of air pollutants during 0–3 days in 4,985 Korean elderly patients with cardiovascular or respiratory disease [3]. Emergency department visits with depressive disorders and suicide attempts showed associations with CO, NO_2_, SO_2_, and PM_10_ in 27,047 Canadians [4, 5]. However, these associations were different according to seasons [6] or represent only sort-term exposure to air pollutants. Although a few studies have addressed the incidence of mental health disorders in patients with long-term exposure to air pollution, the association between newly diagnosed major depressive disorder and PM ≤ 2.5μm has been minimally assessed [7]. A study of increased suicide rate after 4 weeks’ exposures to air pollution did not adjust for known risk factors [8]. Moreover, there is inconsistent evidence that ambient air pollution is associated with depressive symptoms among older adults ≥ 65 years of age living in a metropolitan area of U.S. or European general population [9, 10].

In other words, there was insufficient evidence to support an association between long-term exposure to ambient air pollution and mental health status in general population. Therefore, for this study, we used nationwide population data to investigate the association between long-term exposure to ambient air pollution and mental health status, including subjective stress, depressive disorders, health-related quality of life and suicide.

## Materials and methods

### Study participants

For this study, we evaluated data from the Korean Community Health Survey (KCHS) 2013, which has been collected by the Korea Centers for Disease Control and Prevention annually since 2008. We collected the data via computer-assisted personal interviews with 900 people in each of the 253 community units between August and October in 2013, including 17 metropolitan areas and provinces. Study participants aged 19 or older in each area who were selected by the probability proportional sampling method and the systematic sampling method [11]. Among total surveyed 228,781 adults in 2013, we selected subjects who had lived in the same domicile for > five years. After we matched the domicile code of participants and the location code of air pollution surveillance station because of using same code system, we ultimately analyzed 124,205 persons (unweighted number).

### Air pollutant variables

We obtained the daily average concentrations of hourly measured particulate matter <10 μm (PM_10_), NO_2_, CO, and SO_2_ as air pollutant variables at nationwide air pollution surveillance stations from the Korean Air Pollutants Emission Service. We calculated quartiles of air pollutants using yearly average concentration between August 2012 and July 2013. These air pollutant measurements followed the standard reference protocol of the Korean Air Pollutants Emission Service [12]. PM_10_ had been measured using beta-ray attenuation method (MEZUS-610, KENTEK, Daejeon, Korea). NO_2_ had been measured using chemiluminescence method (MEZUS-210, KENTEK). CO had been measured using non-dispersive infrared (MEZUS-310, KENTEK). SO_2_ had been measured using UV fluorescence (MEZUS-110, KENTEK). We obtained meteorological data, including temperature, rainfall, and wind speed, from the National Meteorological Office in the same period [13, 14].

### Mental health variables

The KCHS surveyed mental health-related indicators. These indicators were defined as subjective daily stress, the EuroQol-5 dimensions (EQ-5D) index, the presence or absence of depressiveness (such as a feeling of sadness or hopelessness lasting more than two consecutive weeks), physician’s diagnosis of depression, suicidal ideation, or a suicide attempt during the past year. We assessed subjective stress on a four-point rating scale (“very much,” “a lot,” “a little bit,” “rarely”). Ultimately, we defined participants with subjective stress as those who responded with “very much” or “a lot” of stress. The EQ-5D index is broadly applied to evaluate health-related quality of life in five dimensions (mobility, self-care, usual activities, pain/discomfort, and anxiety/depression), and each dimension has one of three possible responses (no problems, some problems, or extreme problems). The EQ-5D index generates a single value from each dimension using the following weighted health scores: worst possible = 0; best possible = 1, and a score below zero equates to a health status worse than death [15]. We defined the fourth quartile of the EQ-5D index (which was 0.913 in this study) as a group with poor quality of life.

### Other variables

We categorized patients as non-smokers, former smokers (smoked at one time but not currently), or current smokers (smoking daily or intermittently at the time of the survey). We defined alcohol consumption by drinking frequency of one time per week. We defined physical activity by intensity and frequency. Active group was doing moderate intense activity ≥ three times per week or vigorous activity ≥ one time per week. Inactive group was defined when participant was not met these criteria. Vigorous physical activity included running (jogging), climbing, fast biking, fast swimming, soccer, basketball, jumping rope, squash, or singles tennis, as well as occupational activities such as carrying heavy objects [16]. We also obtained the following demographic information: years of education (< 9, 9–12, or > 12); marital status (married/with partner, not married, or divorced/widowed); current employment status (employed or retired/unemployed); household income (< 7,000,000 won/year or ≥ 7,000,000 won/year); hours of sleep duration (< 7, 7–9, or > 9); religion (yes or no); residence (rural or urban); and medical history according to physicians’ diagnoses, including hypertension, diabetes mellitus, dyslipidemia, stroke, myocardial infarction, ischemic heart disease, asthma, and arthritis. We divided participants’ length of residence into four groups, 5 ≤ Q1 < 10 years, 10 ≤ Q2 < 15 years, 15 ≤ Q3 < 20 years, or Q4 ≥ 20 years, after excluding those who had lived in their areas for < 5 years.

### Ethical considerations

The institutional review board (IRB) at the Korean Centers for Disease Control and Prevention approved the study protocol, and all of the participants provided written informed consent. The IRB at Gangnam Severance Hospital, Yonsei University College of Medicine approved this study as well (IRB File Number: 3-2017-0153).

### Statistical analyses

We conducted all analyses considering the survey weight. Continuous variables are presented as means with standard errors, and categorical variables are presented as percentages. We conducted a univariate analysis to find out the association between the characteristics of participants and mental health status. We then evaluated mental disorder risk using multiple logistic regression analysis after adjusting for age, sex, smoking, drinking, physical activity, education, marital status, employment, household income, sleep duration, residence, and medical history (hypertension, diabetes mellitus, dyslipidemia, stroke, myocardial infarction, ischemic heart disease, asthma, arthritis). We conducted stratified analyses to investigate the possible effect modification by sex and age (divided by age 65) in subgroup analysis. EQ-5D index was skewed distributed. We showed the meteorological data including mean temperature, rainfall and wind speed and the level of air pollutant in 2013 in S1 Table. The nationwide values of ambient air pollutants are also presented as means with standard deviations, medians and ranges in S1 Table. Therefore, it was analyzed using logarithmic transformation. We conducted all analyses using SAS software 9.4 (SAS Institute Inc., Cary, NC, USA).

## Results

The demographic, socioeconomic characteristics, health-related behaviors, and past medical history of the study population are summarized in **Table 1**. The mean age was 48.2 years, and the study population was 50.1% women. Approximately 70% of participants had lived in the same domicile for > 15 years.

**Table 1:** Baseline characteristics of study population.

Variables	Total	Men	Women
Age, years	48.2±0.04	47.0±0.06	49.4±0.05
Smoking			
Never	61.2	41.6	80.7
Former	16.1	30.9	1.3
Current	22.7	27.5	18.0
Alcohol intake			
Never or less than one time per week	87.1	85.4	88.8
More than one time per week	12.9	15.6	11.2
Physical activity			
Active	44.5	46.2	42.8
Inactive	55.5	53.8	57.2
Education			
< 9 years	22.4	19.1	25.7
9–12 years	31.8	33.0	30.6
> 12 years	45.9	47.9	43.7
Marital status			
Married/with partner	65.0	67.3	62.7
Not married	22.9	26.9	18.9
Divorced/widowed	12.1	5.8	18.4
Employment			
Employed	62.8	77.5	48.2
Retired/unemployed	37.2	22.5	51.8
Household income			
< 7,000,000 won/year	70.0	69.0	70.9
≥7,000,000 won/year	30.0	31.0	29.1
Sleep time, hours			
< 7 hours	48.7	47.6	49.8
7–9 hours	48.1	48.7	47.5
> 9 hours	3.2	3.7	2.7
Religion, yes	28.2	20.6	35.7
Residence of urban	79.7	79.6	79.8
Hypertension	19.5	19.3	19.7
Diabetes mellitus	7.4	8.0	6.9
Dyslipidemia	11.2	10.7	11.7
Stroke	1.4	1.6	1.3
Myocardial infarction	1.0	1.2	0.8
Ischemic heart disease	1.4	1.3	1.5
Asthma	2.4	2.1	2.7
Arthritis	9.6	4.1	15.0
Length of residence			
5–10 years	14.4	13.9	14.9
10–15 years	13.7	13.2	14.2
15–20 years	11.0	10.9	11.1
≥ 20 years	60.8	62.0	59.8
Subjective stress	27.5	32.0	23.0
Poor quality of life	21.7	18.4	32.9
Depressiveness	6.2	4.2	8.0
Depression diagnosis	2.5	1.3	3.7
Suicidal ideation	8.8	6.6	11.3
Suicide attempt	0.4	0.4	0.5

Data was shown by mean and standard error or percentage. Physical active group was defined as moderate intense activity ≥ 3 times per week or vigorous activity ≥1 time per week. Inactive group was not met these criteria. Length of residence with same domicile was counted. Medical history was defined as a physician’s diagnosis. The subjects with subjective stress were defined as those responding with “very much” or “a lot” of stress. The fourth quartile of the EuroQol-5 dimensions index was defined as a group with poor quality of life.

The association between the characteristics of participants and mental health status was shown in **Table 2**. Mental health status was associated with various sociodemographic feature, health-related behaviors and medical factor. The risk of subjective stress decreased older age, education less than 12 years or unemployed participants. Subjects with current smoking and alcohol drinking more than one time per week represented a low risk of depressiveness and depression diagnosis by doctor.

**Table 2:** Univariate analysis for the association between the characteristics of participant and mental health status.

	Subjective stress	Poor quality of life	Depressiveness	Depression diagnosis by doctor	Suicidal ideation	Suicide attempt
Age	0.991 (0.990,0.992)	1.049(1.048,1.051)	1.010 (1.009,1.012)	1.021 (1.019,1.024)	1.024 (1.022,1.025)	1.007 (1.001,1.013)
Women	1.005 (0.977,1.034)	2.325 (2.256,2.396)	1.926 (1.819,2.039)	2.765 (2.523,3.029)	1.731 (1.654,1.811)	1.301 (1.059,1.600)
Current smoking	1.591 (1.539,1.646)	1.713 (1.645,1.784)	0.916 (0.857,0.980)	0.783 (0.707,0.867)	0.996 (0.944,1.051)	2.090 (1.699,2.573)
Alcohol (≥1/week)	1.303 (1.259,1.348)	1.619 (1.555,1.686)	0.880 (0.822,0.943)	0.632 (0.566,0.705)	0.951 (0.900,1.004)	1.570 (1.254,1.967)
Physically inactive	1.101 (1.068,1.134)	1.757 (1.700,1.815)	1.122 (1.061,1.187)	1.381 (1.270,1.502)	1.323 (1.264,1.386)	1.454 (1.179,1.974)
Education, ≤ 12 years	0.939 (0.911,0.967)	2.818 (2.720,2.921)	1.594 (1.502,1.692)	2.280 (2.070,2.511)	2.273 (2.155,2.398)	3.100 (2.414,3.980)
Divorced/widowed	1.091 (1.057,1.125)	1.324 (1.282,1.368)	1.405 (1.330,1.485)	1.395 (1.284,1.516)	1.309 (1.250,1.371)	1.589 (1.297,1.947)
Unemployed	0.728 (0.706,0.750)	2.949 (2.858,3.044)	1.759 (1.665,1.859)	2.784 (2.566,3.020)	1.736 (1.659,1.816)	2.010 (1.638,2.468)
Household income < 7,000,000	1.077 (1.038,1.117)	1.714 (1.643,1.788)	1.453 (1.349,1.564)	1.728 (1.542,1.936)	1.608 (1.509,1.713)	2.312 (1.693,3.158)
Sleep time <7,or ≥9	1.518 (1.474,1.564)	1.443 (1.399,1.488)	1.493 (1.412,1.579)	1.586 (1.461,1.721)	1.509 (1.443,1.579)	1.952 (1.584,2.405)
Residence of urban	1.030 (0.989,1.073)	0.795 (0.761,0.829)	1.083 (1.005,1.167)	1.069 (0.949,1.204)	0.847 (0.797,0.900)	0.888 (0.686,1.149)
Hypertension	0.962 (0.918,1.018)	2.905 (2.810,3.003)	1.381 (1.299,1.469)	1.883 (1.731,2.049)	1.793 (1.708,1.882)	1.559 (1.246.1.950)
Diabetes Mellitus	1.023 (0.969,1.079)	2.754 (2.625,2.888)	1.512 (1.390,1.645)	2.056 (1.829,2.310)	1.956 (1.828,2.093)	2.205 (1.636,2.973)
Dyslipidemia	1.175 (1.125,1.228)	2.170 (2.082,2.261)	1.658 (1.546,1.779)	2.747 (2.511,3.006)	1.776 (1.674,1.885)	1.614 (1.260.2.068)
Stroke	1.316 (1.182,1.465)	9.142 (8.160,10.243)	2.523 (2.172,2.931)	3.513 (2.941,4.197)	3.402 (3.024,3.828)	4.156 (2.718,6.353)
Myocardial infarction	1.158 (1.020,1.316)	4.391 (3.903,4.940)	2.297 (1.938,2.723)	2.730 (2.158,3.453)	2.733 (2.354,3.172)	3.237 (1.847,5.675)
Ischemic heart disease	1.142 (1.025,1.272)	4.276 (3.879,4.713)	2.195 (1.874,2.571)	3.885 (3.267,4.621)	2.712 (2.397,3.069)	2.344 (1.357,4.049)
Asthma	1.495 (1.368,1.635)	2.844 (2.615,3.094)	2.629 (2.327,2.970)	3.073 (2.594,3.642)	2.763 (2.498,3.055)	2.554 (1.681,3.882)
Arthritis	1.219 (1.165,1.276)	7.433 (7.114,7.767)	2.440 (2.279,2.613)	3.747 (3.434,4.088)	2.949 (2.789,3.118)	2.405 (1.876,3.083)

Physically inactive group was defined when participant was doing moderate intense activity < three times per week or vigorous activity < one time per week; years of education (≤ 12, or > 12); marital status (married/with partner, not married, or divorced/widowed); current employment status (employed or retired/unemployed); household income (< 7,000,000 won/year or ≥ 7,000,000 won/year); hours of sleep duration (7–9, and <7 or > 9); residence (rural or urban); and medical history according to physicians’ diagnoses, including hypertension, diabetes mellitus, dyslipidemia, stroke, myocardial infarction, ischemic heart disease, asthma, and arthritis.

**Fig 1:**
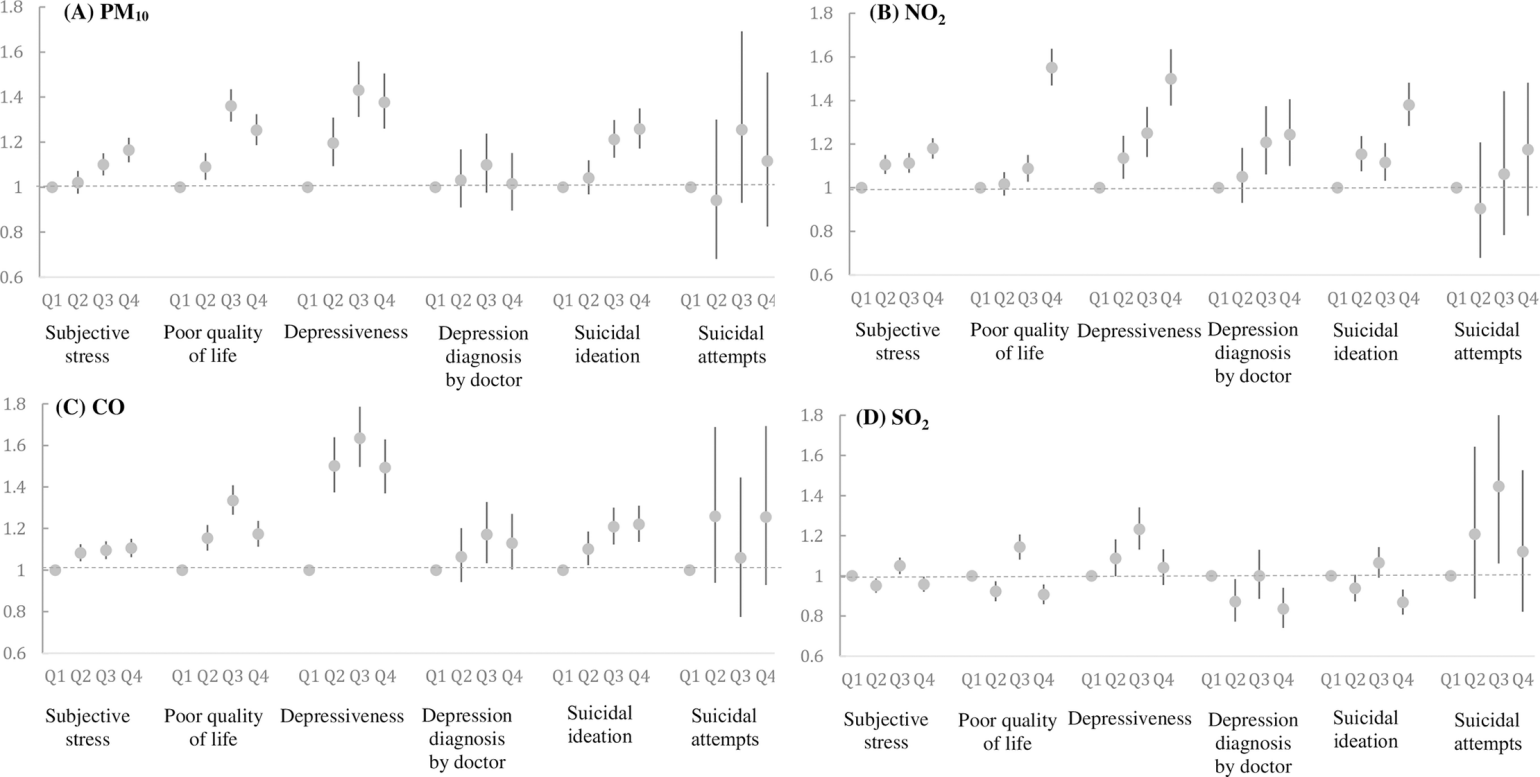
The odds ratios and 95% confidence intervals of a mental health disorder according to the air pollutant quartile. (A) PM_10_ (B) NO_2_ (C) CO (D) SO_2_.

The risk of a mental disorder according to the air pollutant quartile is represented in **Fig 1**. After we adjusted for confounding factors, there were positive associations between PM_10_, NO_2_, CO exposure and mental health status except suicidal attempts. The risk of depressiveness increased at the third quartile of CO exposure (odds ratio [OR]; 95% confidence interval [CI]: 1.635(1.497, 1.786)), the highest quartile of NO_2_ (1.501(1.377, 1.635)) and the third quartile of PM_10_ (1.335(1.267, 1.408)). There was no association between SO_2_ exposure and mental health status.

Compared with women, men had increased prevalence of subjective stress with exposure to PM_10_ and prevalence of poor QoL with exposure to CO and SO_2_ in **Table 3**. And depressiveness in men also increased with exposure to NO_2_, CO and SO_2_. The risk of depression diagnosis by doctor and suicidal ideation had no difference according to sex (*Ps* > 0.05). The effect of SO_2_ was inconsistent according to the quartiles.

**Table 3:** Air pollution and mental health status according to sex.

	Subjective stress		Poor quality of life		Depressiveness		Depression diagnosis by doctor		Suicidal ideation	
	Men	Women	Men	Women	Men	Women	Men	Women	Men	Women
**PM**_**10**_										
Q4	**1.121(1.062, 1.183)**	**1.089(1.036,1.145)**	1.256(1.175,1.337)	1.236(1.137, 1.344)	1.342(1.158,1.556)	1.385(1.248,1.536)	0.978(0.758,1.263)	1.022(0.886,1.179)	1.241(1.109,1.388)	1.256(1.154,1.368)
Q3	**1.078(1.028,1.131)**	1.033(0.981, 1.087)	1.360(1.278,1.446)	1.338(1.233, 1.451)	1.442(1.250,1.662)	1.409(1.275,1.557)	1.076(0.849,1.363)	1.101(0.960,1.262)	1.246(1.117,1.391)	1.175(1.081,1.277)
Q2	**1.005(0.955,1.058)**	0.998(0.945, 1.053)	1.088(1.020,1.159)	1.078(0.989, 1.175)	1.208(1.040,1.403)	1.184(1.066,1.314)	1.034(0.804,1.331)	1.026(0.890,1.182)	1.013(0.899,1.141)	1.051(0.965,1.144)
Q1	1	1	1	1	1	1	1	1	1	1
p-inter action	**0.009**		0.593		0.741		0.969		0.583	
**NO**_**2**_										
Q4	1.205(1.140, 1.274)	1.161(1.104,1.220)	1.587(1.458, 1.727)	1.518(1.426,1.617)	**1.707(1.479,1.970)**	**1.389(1.252,1.542)**	1.280(1.010, 1.623)	1.223(1.066,1.403)	1.319(1.177,1.478)	1.402(1.286,1.529)
Q3	1.119(1.057, 1.184)	1.108(1.053,1.167)	1.140(1.042, 1.274)	1.057(0.989,1.129)	**1.440(1.238,1.675)**	**1.158(1.038,1.291)**	1.125(0.879, 1.440)	1.241(1.069,1.441)	1.074(0.957,1.205)	1.134(1.031,1.247)
Q2	1.139(1.079, 1.202)	1.072(1.021,1.126)	1.054(0.972, 1.142)	0.996(0.937,1.059)	**1.146(0.991,1.325)**	**1.128(1.017,1.251)**	1.005(0.783, 1.290)	1.059(0.929,1.208)	1.045(0.943,1.158)	1.197(1.098,1.304)
Q1	1	1	1	1	1	1	1	1	1	1
p-inter action	0.054		0.391		**0.011**		0.765		0.205	
**CO**										
Q4	1.123(1.064, 1.186)	1.091(1.038,1.147)	**1.196(1.123,1.274)**	**1.135(1.045, 1.232)**	**1.535(1.338,1.663)**	**1.524(1.375,1.689)**	1.204(0.946, 1.533)	1.100(0.964,1.256)	1.318 (1.178,1.476)	1.162(1.069,1.263)
Q3	1.111(1.051, 1.173)	1.085(1.032,1.140)	**1.433(1.346,1.526)**	**1.186(1.091, 1.290)**	**1.697(1.465,1.966)**	**1.584(1.424,1.763)**	1.091(0.852, 1.397)	1.197(1.039,1.378)	1.290(1.148, 1.450)	1.152(1.057, 1.256)
Q2	1.089(1.032, 1.150)	1.079(1.027,1.134)	**1.212(1.139,1.290)**	1.065(0.980, 1.158)	**1.389(1.176,1.593)**	**1.369(1.112,1.643)**	0.999(0.776, 1.286)	1.086(0.951,1.241)	1.163(1.034, 1.307)	1.061(0.975, 1.155)
Q1	1	1	1	1	1	1	1	1	1	1
p-inter action	0.580		**<0.001**		**0.045**		0.633		0.324	
**SO**_**2**_										
Q4	0.944(0.894, 0.997)	0.973(0.925,1.023)	0.909(0.852,0.970)	0.905(0.835,0.981)	1.146(0.933,1.323)	0.986(0.891,1.091)	0.944(0.738, 1.208)	0.804(0.701,0.922)	0.879(0.785,0.984)	0.861(0.791,0.938)
Q3	1.042(0.986, 1.101)	1.059(1.008,1.114)	**1.145(1.074,1.220)**	**1.129(1.037,1.229)**	**1.345(1.162,1.557)**	**1.171(1.059,1.294)**	1.082(0.848, 1.379)	0.977(0.849,1.125)	1.091(0.972,1.224)	1.045(0.959,1.139)
Q2	0.939(0.890, 0.991)	0.962(0.917,1.010)	0.972(0.912,1.035)	0.851(0.783,0.925)	1.013(0.876,1.172)	1.131(1.025,1.248)	1.018(0.797, 1.302)	0.834(0.729,0.955)	0.909(0.812,1.018)	0.962(0.885,1.047)
Q1	1	1	1	1	1	1	1	1	1	1
p-inter action	0.738		**0.017**		**0.002**		0.527		0.488	

Bold characteristics means *P*–value <0.05 among the values with *p*-interaction < 0.05. EQ-5D index was analyzed by logarithmic transformation. Adjustment for age, smoking, drinking, physical activity, education, marital status, employment, household income, sleep duration, residence and medical history (hypertension, diabetes mellitus, dyslipidemia, stroke, myocardial infarction, ischemic heart disease, asthma, arthritis).

The risk of higher stress and poor QoL with PM_10_ in subjects < age 65 were significantly increased than that in subjects ≥ age 65 in **Table 4**. Subjects < age 65 with high quartiles of PM_10_, NO_2_, CO and SO_2_ had a higher risk of poor QoL than subjects ≥ age 65. In the higher levels of air pollutants, the risk of depressiveness, depression diagnosis by doctor and suicidal ideation increased, however, there had no significant difference according to age 65.

**Table 4:** Air pollution and mental health status according to age.

	Subjective stress		Poor quality of life		Depressiveness		Depression diagnosis by doctor		Suicidal ideation	
	Age ≥ 65	Age < 65	Age ≥ 65	Age < 65	Age ≥ 65	Age < 65	Age ≥ 65	Age < 65	Age ≥ 65	Age < 65
**PM**_**10**_										
Q4	0.940(0.872, 1.014)	**1.150(1.101,1.201)**	0.949(0.867, 1.038)	**1.338(1.277,1.456)**	1.329(1.137,1.555)	1.383(1.246,1.535)	0.866(0.707,1.059)	1.061(0.910,1.237)	1.192(1.067, 1.331)	1.269(1.165,1.382)
Q3	0.986(0.918, 1.060)	**1.062(1.018,1.108)**	**1.101(1.012, 1.198)**	**1.473(1.383,1.569)**	1.327(1.142,1.542)	1.446(1.310,1.600)	0.907(0.752,1.049)	1.154(0.996,1.338)	1.211(1.094, 1.341)	1.206(1.108,1.312)
Q2	0.919(0.854, 0.988)	1.025(0.979,1.073)	1.006(0.926, 1.094)	**1.122(1.049,1.201)**	1.189(1.028,1.376)	1.200(1.079,1.334)	1.027(0.856,1.232)	1.022(0.874,1.194)	1.054(0.945, 1.175)	1.033(0.945,1.130)
Q1	1	1	1	1	1	1	1	1	1	1
p-inter action	**<0.001**		**<0.001**		0.688		0.218		0.765	
**NO**_**2**_										
Q4	1.182(1.095,1.277)	1.171(1.120,1.225)	**1.203(1.101,1.314)**	**1.706(1.598,1.821)**	1.550(1.341,1.792)	1.478(1.337,1.633)	1.071(0.884,1.299)	1.289(1.107,1.501)	1.319(1.177,1.478)	1.702(1.286,1.529)
Q3	1.189(1.101,1.284)	1.093(1.044,1.144)	0.843(0.771,0.923)	**1.207(1.127,1.294)**	1.334(1.144,1.556)	1.223(1.098,1.362)	1.123(0.929,1358)	1.231(1.049,1.444)	1.074(0.957,1.205)	1.134(1.031,1.247)
Q2	1.096(1.023,1.174)	1.109(1.061,1.160)	0.932(0.861,1.009)	1.068(0.999,1.141)	1.101(0.963,1.258)	1.139(1.027,1.262)	0.974(0.818,1.153)	1.080(0.929,1.254)	1.045(0.943,1.158)	1.197(1.098,1.304)
Q1	1	1	1	1	1	1	1	1	1	1
p-inter action	0.060		**<0.001**		0.662		0.893		0.181	
**CO**										
Q4	1.091(1.014,1.173)	1.111(1.063,1.162)	0.971(0.890,1.061)	**1.249(1.172,1.332)**	1.626(1.398,1.891)	1.463(1.322,1.618)	1.213(1.006,1.462)	1.110(0.961,1.284)	1.115(0.999, 1.244)	1.255(1.152,1.368)
Q3	1.060(0.984,1.142)	1.098(1.051,1.148)	0.999(0.916,1.090)	**1.470(1.379,1.567)**	1.600(1.368,1.872)	1.635(1.475,1.811)	1.094(0.898,1.331)	1.187(1.020,1.382)	1.145(1.027, 1.277)	1.229(1.125,1.343)
Q2	1.097(1.019,1.182)	1.069(1.025,1.116)	0.997(0.913,1.089)	**1.234(1.157,1.316)**	1.473(1.260,1.721)	1.504(1.359,1.666)	1.103(0.905,1.343)	1.050(0.905,1.218)	1.027(0.920, 1.147)	1.131(1.035,1.236)
Q1	1	1	1	1	1	1	1	1	1	1
p-inter action	0.758		**<0.001**		0.329		0.602		0.265	
**SO**_**2**_										
Q4	0.994(0.921,1.072)	0.955(0.914,0.998)	0.934(0.855,1.022)	0.902(0.846,0.961)	1.272(1.094,1.479)	0.987(0.894,1.089)	0.977(0.804,1.187)	0.901(0.775,1.043)	0.947(0.850,1.056)	0.839(0.771,0.914)
Q3	1.041(0.962,1.126)	1.047(1.002,1.095)	1.025(0.934,1.125)	**1.181(1.109,1.258)**	1.341(1.149,1.563)	1.203(1.091,1.326)	0.987(0.817,1.191)	1.195(0.944,1.271)	1.022(0.916,1.141)	1.074(0.986,1.169)
Q2	0.957(0.889,1.030)	0.947(0.907,0.989)	0.995(0.910,1.086)	0.908(0.851,0.969)	1.193(1.027,1.386)	1.066(0.968,1.175)	0.954(0.781,1.166)	0.935(0.805,1.085)	0.934(0.841,1.038)	0.942(0.865,1.026)
Q1	1	1	1	1	1	1	1	1	1	1
p-inter action	0.500		**<0.001**		0.092		0.825		0.104	

Bold characteristics means *P*–value <0.05 among the values with *p*-interaction < 0.05. EQ-5D index was analyzed by logarithmic transformation. Adjustment for sex, smoking, drinking, physical activity, education, marital status, employment, household income, sleep duration, residence and medical history (hypertension, diabetes mellitus, dyslipidemia, stroke, myocardial infarction, ischemic heart disease, asthma, arthritis)

## Discussion

In this study, we used Korean nationwide population-based data to identify associations between long-term exposure to ambient air pollutants and mental health status. After considering mental health-related confounding factors such as socioeconomic status, health-related behavior and medical history, air pollutants may be an independent predictor of mental health status, ranging from subjective stress level to suicidal ideation.

Our results were similar to those of a previous Korean study in which emergency department visits for depressive episodes in patients with a past history of depressive disorder were associated with recent air pollutant levels [3]. However, our study findings confirmed the associations between subjective stress in daily life or suicide attempts in the general population and long-term exposure to ambient air pollutants. In a three-year study from the National Health Insurance database, there was an association between major depressive disorder and PM_2.5_ [7]. However, we additionally assessed the effects of SO_2_, NO_2_, and CO on mental health status, and thereby, we confirmed the associations between long-term exposure to ambient air pollutants including PM_10_, NO_2_, and CO and subjective stress, poor QoL, depressiveness, and suicide ideation.

In this study, we found no clear linear correlation between the risk of mental health disorders and the air pollutant concentration quartile. We believe that the reason for this finding is a threshold effect at low levels of air pollutants; if the concentration is above a certain cut-off value, a significant effect may be similar. We also identified a weak association between depression diagnosis by a physician and ambient air pollution quartile. This association may decrease after adjustment of the known risk factors for depression diagnosis, but a strong association between air pollution and parameters of other mental health status was maintained. Therefore, air pollution may be an unknown risk factor in other mental health parameters. In addition, undiagnosed depressive patients may have other risk factors. In generally, it was known that the risk of mental health disorder was higher in women and the elderly, but air pollution may be an important risk factor for men or persons < 65 years old because these groups may be exposed to air pollution more frequently with high activity [17, 18]. Except the rate of subjective stress, women’s mental health status showed more poor than men in this study, though the rates of suicide attempt were similar. It has been proposed that men’s mental health status may be masked by alcohol and physical violence, and their diagnosis of depression may be underdiagnosed [19]. Accordingly, known confounding factors may be correlated with women’s diagnosed depression from the previous studies [19]. Therefore, air pollutants, as a new association factor of mental health status, may be found out an independent risk factor and enhanced the risk for men. Further research is needed to support any such causal relationship or biological difference. In this study, there was no association between suicide attempts and air pollution exposure. Suicide attempts represent acute symptom worsening, which may be more influenced by short-term rather than long-term exposure to ambient air pollutants [5, 20].

Air pollutants may be strong inflammatory agents in psycho-endocrine-immune connections through an inflammatory process; cyclooxygenase-2, interleukin-1β and particulate-matter–associated lipopolysaccharides [21]. Exposure to air pollutants leads to elevated hippocampal pro-inflammatory cytokine expression, and in addition, there are architectural changes in the dendrites of the hippocampus that can increase depressive-like behaviors in animal models [22]. Neuroinflammation caused by exposure to air pollution can alter innate immune responses and even influence human neurodegenerative disease [21].

Particulate and gaseous pollutants coexist in the air and may induce adverse health effects. PM rarely exists by itself within the ambient environment because gaseous, semi-volatile, and volatile compounds (i.e., aldehydes and polycyclic aromatic hydrocarbons) are constantly changing and interacting. Many vapor-phase compounds attach to the surface of PM and/or by themselves form secondary aerosolized particles [1]. The concentrations of PM_10_ and NO_2_ are highly correlated because they share the same pathway as that for depression and neurologic disorders [23]; in contrast, NO_2_ and SO_2_ may have different physiologic influences on human health. Although both are gaseous molecules, NO_2_ is poorly soluble in water, whereas SO_2_ is highly water soluble [24].

This study has a number of strengths. For one, we used large, heterogeneous, nationwide population-based data. We also considered a wide range of known covariates related to depression, including socioeconomic status [25]. Therefore, we were able to determine the effects of ambient air pollution as an independent risk factor for poor mental health. In particular, previously known risk factors were related to women and the elderly, but this study confirmed that air pollution was a risk factor for mental health disorders in men and individuals < 65 years old.

This study also has several limitations. First, it was not possible to establish causality between air pollution and mental health disorders in this cross-sectional study. Second, we matched the community and air pollutant levels using participants’ home territories. Therefore, if participants worked far from their domiciles, the matching would not have accurately reflected air pollutant levels at their dwellings. Third, we focused on each pollutant and its respective effect on mental health status. However, the adverse effects of pollution on mental health may be caused by unmeasured pollutants, such as PM_2.5_ or ozone [26], or combinations of multiple pollutants [6], which the effects of complex mixtures of constituent toxins on mental health cannot be explained in this study. Additionally, KCHS did not include the surveyed day to protect personal information, which linked each other. Weather condition may affect the level of air pollution and human activity, however, we cannot adjust them [13]. However, KCHS surveyed during three months, which had stable weather conditions from August to October without monsoon. Therefore, the weather effect would be relatively minimized. Lastly, the prevalence of depression in this study (2.49%) was lower than that found in previous studies of Koreans (3.7%) and Canadians (3.9%) [27, 28]. Therefore, we may have underestimated the prevalence of mental health disorders in these nationwide population-based KCHS data.

## Conclusions

Long-term exposure to ambient air pollution was a risk factor of a wide range of potential mental health disorders. Future investigations must not only include more studies to determine the mechanisms of action but also examine the effect of demographic characteristics. This information is helpful to make a policy to control air pollution and to understand the action of air pollutant on the body correctly.

## Supporting information

S1 Table The ambient air pollutants and meteorological data in Korea. Particulate matter <10 μm (PM_10_); Sulfur dioxide (SO_2_); Nitrogen dioxide (NO_2_); Carbon monoxide (CO). Temperature, rainfall and wind speed were shown at Seoul (Lat.(N) 37°34´, Long.(E) 126°57´). Korea Meteorological Administration, Seoul, Korea (Aug. 2012- July. 2013) http://www.kma.go.kr/repositary/sfc/pdf/sfc_ann_2013.pdf.
(DOCX)
