# Supplementary material for: Long-term exposure to ambient air pollutants and mental health status: A nationwide population-based cross-sectional study
Source: PLoS One. 2018 Apr 9;13(4):e0195607. doi: 10.1371/journal.pone.0195607 (PMC5891065; doi:10.1371/journal.pone.0195607)
Supplement: S1 Table — Particulate matter <10 μm (PM10); Sulfur dioxide (SO2); Nitrogen dioxide (NO2); Carbon monoxide (CO). Temperature, rainfall and wind speed were shown at Seoul (Lat.(N) 37°34´, Long.(E) 126°57´). Korea Meteorological Administration, Seoul, Korea (Aug. 2012- July. 2013) http://www.kma.go.kr/repositary/sfc/pdf/sfc_ann_2013.pdf. (DOCX) [file pone.0195607.s001.docx]

**S1 Table**. **The ambient air pollutants and meteorological data in Korea**

|  | Mean ± Standard deviation | Median | Range |
| --- | --- | --- | --- |
| PM_10_ (μg/m^3^) | 48.6±8.3 | 47.5 | 31.4-72.5 |
| SO_2_ (ppb) | 5.6±1.9 | 5.3 | 2.4-16.2 |
| NO_2_ (ppb) | 24.0±7.8 | 23.6 | 8.3-42.7 |
| CO (10 ppm) | 5.3±1.1 | 5.4 | 2.7-9.5 |
| Temperature (̊C) | 12.4±11.1 | 13.8 | -4.1~27.7 |
| Rainfall (mm) | 110.4±155.2 | 58.4 | 0.8~676.2 |
| Wind speed (m/s) | 2.8±0.4 | 2.7 | 2.2~3.5 |

Particulate matter <10 μm (PM_10_); Sulfur dioxide (SO_2_); Nitrogen dioxide (NO_2_); Carbon monoxide (CO). Temperature, rainfall and wind speed were shown at Seoul (Lat.(N) 37˚34´, Long.(E) 126˚57´). Korea Meteorological Administration, Seoul, Korea (Aug. 2012- July. 2013) <http://www.kma.go.kr/repositary/sfc/pdf/sfc_ann_2013.pdf>
